# Agarose Film-Based Liquid–Solid Conversion for Heavy Metal Detection of Water Samples by Laser-Induced Breakdown Spectroscopy

**DOI:** 10.3390/molecules28062777

**Published:** 2023-03-19

**Authors:** Zhengkai You, Xiaolong Li, Jing Huang, Rongqin Chen, Jiyu Peng, Wenwen Kong, Fei Liu

**Affiliations:** 1 College of Biosystems Engineering and Food Science, Zhejiang University, Hangzhou 310058, China; 2 College of Mechanical Engineering, Zhejiang University of Technology, Hangzhou 310023, China; 3 College of Mathematics and Computer Science, Zhejiang A&F University, Hangzhou 311300, China

**Keywords:** heavy metal detection, laser-induced breakdown spectroscopy, liquid–solid conversion, agarose film

## Abstract

Laser-induced breakdown spectroscopy (LIBS) shows promising applications in the analysis of environmental heavy metals. However, direct analysis in water by LIBS faces the problems of droplet splashing and laser energy decay. In this study, a novel liquid–solid conversion method based on agarose films is proposed to provide an easy-to-operate and sensitive detection of heavy metals. First, the water samples were converted into semi-solid hydrogels with the aid of agarose and then dried into agarose films to make the signal intensities stronger. The calibration curves of Cd, Pb and Cr were constructed. The proposed method was validated by standard heavy metal solutions and real water samples. The results showed that the values of R^2^ were 0.990, 0.989 and 0.975, and the values of the LOD were 0.011, 0.122 and 0.118 mg L^−1^ for Cd (I) 228.80, Pb (I) 405.78 and Cr (I) 427.48 nm, respectively. The RMSEs of validation were 0.068 (Cd), 0.107 (Pb) and 0.112 mg·L^−1^ (Cr), and the recovery values were in the range of 91.2–107.9%. The agarose film-based liquid–solid conversion method achieved the desired ease of operation and sensitivity of LIBS in heavy-metal detection, thereby, showing good application prospects in heavy metal monitoring of water.

## 1. Introduction

As the continuation of human activities and industrialization, various untreated pollutants are discharged into the environment [1]. According to the World Health Organization (WHO) and United States Environmental Protection Agency (USEPA), the average concentration (1972–2017) of heavy metals in global rivers and lakes has exceeded the threshold limits [2]. Heavy metal pollution has become a major global concern [3]. 

The severe heavy metal pollution poses a great threat not only to the ecological environment but also to human health. Heavy metals can diffuse in the environment and reach the top of the food chain through bioaccumulation mechanisms [4]. Most importantly, they are toxic and cannot be biodegraded [5]. The accumulation of heavy metals in the human body may cause serious damage to the respiratory, digestive systems and central nervous system and is extremely carcinogenic [6,7,8].

Due to the widespread contamination and serious harm of heavy metals in water, the monitoring of heavy metal pollution in water is receiving increasing attention. Atomic absorption spectroscopy (AAS), inductively coupled plasma mass spectroscopy (ICP-MS) and ultraviolet-visible spectrophotometry (UV-VIS) are the traditional methods used for heavy metal detection in water samples [9]. However, these methods often need complex sample preparation processes and high technical requirements for the operators. With the advantages of simple sample preparation process as well as fast and direct detection compared to conventional analytical techniques, laser-induced breakdown spectroscopy (LIBS) has been extensively studied for elemental detection [10].

LIBS is an atomic emission spectroscopy technique based on high-energy laser sampling [11]. After irradiating the sample with high-energy density pulsed lasers, the plasma emission spectra containing information on the type and content of the elements of the sample to be measured is produced. The qualitative and quantitative determination of the sample elements can be achieved by analytical plasma spectra. Theoretically, under the action of extremely high laser energy, substances can be ablated into a plasma state; thus, the LIBS technique can be used to analyze samples in any phase, including gases, liquids and solids [12]. Researchers have made great efforts in theoretical exploration and engineering application of LIBS [13]. Thus far, LIBS has been applied in industrial production [14], geological exploration [15], environmental analysis [16] and many other fields.

Water samples are an important category for LIBS analysis. However, there are some drawbacks when LIBS is applied directly to water samples. On the one hand, when the laser pulse is focused on the surface of the water body, the huge impact generated by the laser is likely to lead to droplet splashing and contamination of the optical path, and the ripples on the water surface will interfere with the coupling between the laser and the sample, thus, affecting the detection accuracy. On the other hand, a large part of the laser energy is used for the vaporization of water, which greatly reduces the efficiency of the laser energy utilization, resulting in a significant decrease in the intensity of the plasma emission spectrum and affecting the sensitivity of detection [17].

In order to achieve accuracy and sensitivity in elemental detection of water samples, the moisture effect of the LIBS detection process needs to be reduced. Water sample introduction systems were designed to change the morphology of the water and reduce droplet splash, including liquid jets [18], droplet [19] and atomization [20]. However, laser energy loss and water splash in LIBS detection still exist, resulting in weak spectral intensity [21]. In addition, the systems are not convenient to install on LIBS and are troublesome to clean after each use. 

In order to solve these problems, liquid–solid conversion has been investigated to eliminate the drawbacks of LIBS water detection by transferring the target element from liquid to solid [22] or solidifying the sample [23,24]. The transfer of target elements from liquid to solid can completely eliminate the effects of moisture and can be divided into solid-phase extraction [25] and substrate liquid–solid conversion [26]. The former can obtain high sensitivity benefiting from the high enrichment ratio. However, absorbent materials are often expensive and require precise control of the extraction conditions, such as the temperature, time and pH, which have a significant impact on the extraction effect, which, in turn, affects the accuracy of the detection. The latter, which involves the transfer of the target elements from a water sample to a solid substrate surface, has become a popular strategy for the advantages of convenient operation and enrichment of elements [27,28]. However the evaporation process of water droplets is often accompanied by the coffee ring effect that greatly affects the uniformity of sediment distribution and affects the detection accuracy [27,29]. 

The existing solidification method suppresses the droplet splash and water surface fluctuation during laser-induced breakdown by reducing the mobility of the water sample and achieves better reproducibility and accuracy compared with the LIBS direct water-detection method. However, the presence of moisture results in a weak spectral signal that cannot meet the needs of LIBS for the detection of trace heavy metals in water.

To improve the easy-to-operate and sensitive LIBS water sample detection, a new liquid–solid conversion method based on agarose films was proposed. Agarose is a polysaccharide compound isolated from seaweed, which has the property of forming a good semi-solid hydrogel when mixing with water and heating to boiling point and then cooling [30]. The simple composition and thermal gelability of agarose allow it to be used as an ideal water solidifying aid. In this new liquid–solid conversion method, to reduce the water splashing and surface ripples, liquid samples were converted into hydrogels with the help of the thermal gelation properties of agarose. To meet the need for high sensitivity detection, agarose hydrogels were dried into agarose films containing heavy metals to further eliminate the detrimental effects of moisture on LIBS detection.

The main objectives of this work were (1) to compare the LIBS emission spectral intensity and the detection sensitivity of target heavy metals using agarose hydrogels and agarose films; (2) to find the reasons for the difference in LIBS spectra between agarose hydrogels and agarose films by analyzing the ablation crater morphology parameters; (3) to optimize the main parameters affecting agarose film-based liquid–solid method of the agarose-to-solution ratio, delay time and the laser pulse energy; and (4) to achieve high sensitivity for the quantitative detection of Cd, Pb and Cr in water by establishing calibration curves.

## 2. Results and Discussion

### 2.1. Sample Comparison

#### 2.1.1. Spectral Intensity

**Figure 1:**
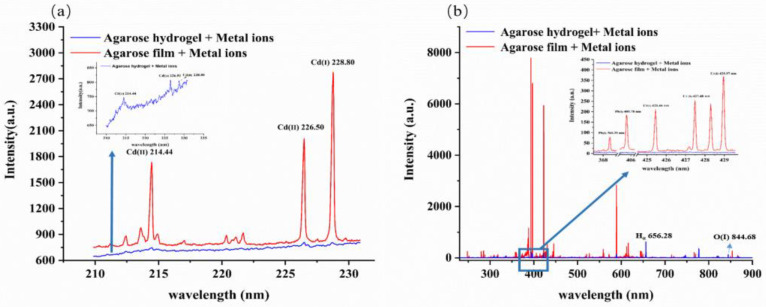
The LIBS spectra of agarose hydrogel and agarose film samples prepared by 2 mg L^−1^ metal ion solution collected by spectrometer SR-500i-A-R (**a**) and spectrometer ME5000 (**b**).

In this study, agarose hydrogel and agarose film samples prepared by 2 mg L^−1^ heavy metal solutions were detected under same experimental conditions to compare the emission spectra. Figure 1 shows the comparative spectra collected by the spectrometer SR-500i-A-R and the spectrometer ME5000. We found that most of the blue lines from agarose hydrogel samples could not be observed clearly. However, red lines from agarose film samples had distinct lines. The results indicated that high moisture content could cause a significant decrease, which was consistent with previous research [31,32]. However, the H and O lines in blue were higher than those in red because the intensities of the H and O lines were positively correlated with the water content [33], and agarose hydrogel sample had a higher water content than did the agarose film samples.

Among the agarose hydrogel samples containing the three heavy metal elements to be measured, only the characteristic spectral lines of Cd (Cd (II) 214.44 nm, Cd (II) 226.50 nm and Cd (I) 228.80 nm) could be vaguely distinguished, while the characteristic spectral lines of Pb (Pb (I) 368.35 nm and Pb (I) 405.78 nm) and Cr (Cr (I) 425.43 nm, Cr (I) 427.48 nm and Cr (I) 428.97 nm) were concealed by the background signal. However, the elemental spectral lines in the agarose film sample could be effectively excited with strong signal intensities. This indicates that the presence of moisture in the sample was the main reason for the weakened signal intensity. 

When the agarose hydrogel sample was irradiated by a laser pulse, part of the laser energy was used first for the evaporation of water. Subsequently, the water vapor dissociated and ionized, resulting in laser energy shielding [31]. In addition, too low concentrations of the target elements in the agarose hydrogel samples may also result in the failure to excite the corresponding emission spectra. In the agarose film sample, due to the removal of water, on the one hand, the laser energy was fully used for the ablation of the sample, and on the other hand, it also had a concentrating effect on the target elements in the sample, which increased the concentration of the target elements and made the characteristic spectral intensities of the target elements stronger. Therefore, due to the presence of moisture effects, the transfer of water samples to the gel state alone could not achieve satisfactory detection requirements. Moisture removal after sample hydrogelation was necessary.

#### 2.1.2. Ablation Crater Morphology

**Figure 2:**
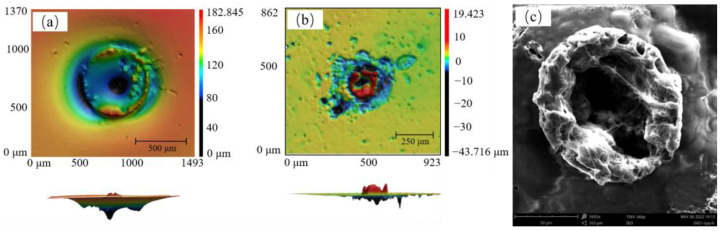
The ablation crater morphology of agarose hydrogel (**a**) and agarose film (**b**); SEM image of ablation crater morphology on agarose film (**c**).

The moisture removal of agarose hydrogel improved the spectral intensities. Ablation crater morphology analysis is useful to understand the performance of laser action on the samples. Figure 2a,b shows the ablation crater morphology of the agarose hydrogels and agarose films using a shape measurement laser microscope system (Keyence VK-X3000) The ablation crater morphology parameters were obtained using the system analysis software. In Figure 2a, the shape of the ablation crater on agarose hydrogel was inverted conical, which was consistent with the ablation crater morphology in most studies [34]. 

At the same time, the outermost layer of the ablation crater was depressed downward because the agarose hydrogel was not a dense structure, and the impact of the high-energy laser caused extrusion on the surface of the sample. For the agarose hydrogel ablation crater, the average ablation crater depth was 53.9 μm, the average cross sectional area was 2.66 × 10^5^ μm^2^, and the average volume of the ablation crater was 1.43 × 10^7^ μm^3^. In Figure 2b, the shape of the ablation crater on agarose film resembles a volcanic crater. The SEM image of the ablation crater on the agarose film sample shows the morphology of the crater after 1650 times magnification in Figure 2c. 

The outer edge of the ablation crater of the agarose film was clearly raised, while the center was slightly shallow. This phenomenon can be explained as follows: when the sample was shot by a high-energy laser beam, in addition to the laser area being ablated, the material at the periphery of the ablation crater was heated and melted, forming a bulge after cooling, similar to solidified magma. This also shows the susceptibility of the agarose film material to ablation. At the same time, due to the dense nature of the agarose film, the laser could not penetrate it. 

The average ablation crater depth was 7.18 μm, the average cross sectional area was 2.23 × 10^3^ μm^2^, and the average volume of the ablation crater was 1.60 × 10^4^ μm^3^. The depth, average cross sectional area and average volume of ablation crater on agarose hydrogel were 7, 120 and 890 times, respectively, than on agarose film. However, for agarose hydrogel samples, a larger amount of ablation did not result in a better detection sensitivity due to the presence of water. Therefore, agarose film-based liquid–solid conversion was chosen for heavy metal detection in the subsequent study.

### 2.2. Characteristic Lines Selection

**Figure 3:**
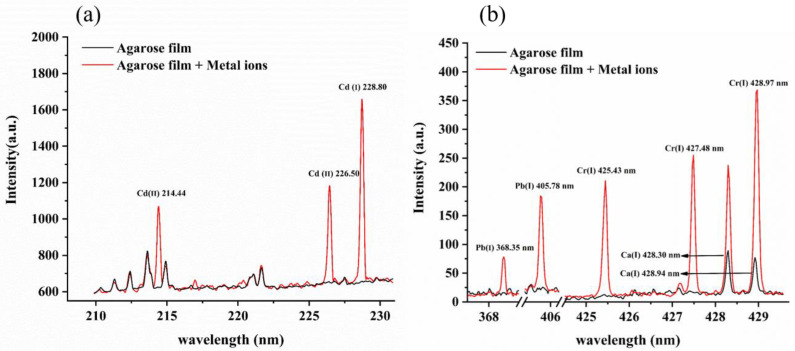
The LIBS spectra of agarose film blank and agarose film samples prepared by 1.25 mg L^−1^ metal ion solution collected by spectrometer SR-500i-A-R (**a**) and spectrometer ME5000 (**b**).

Figure 3a,b show the spectra of agarose film samples containing heavy metals (red line) and blank samples (black line) collected by the spectrometer SR-500i-A-R and spectrometer ME5000, respectively. We found that the blank samples did not contain the target elements (Cd, Pb and Cr); however, the characteristic lines of target elements can be clearly observed in agarose film sample. This showed that the agarose film-based liquid–solid conversion was feasible for the detection of heavy metal elements in water. However, there were quite distinct interference spectral lines near the Cd (I) 214.44 nm. The characteristic peak Cr (I) 428.97 nm could also be influenced by Ca (I) 428.94 nm. There is no doubt that fewer interfering spectral lines and higher intensities of the spectral signal are beneficial for the analysis of the target elements. Considering the need for the sensitive detection of elements, in this study, the characteristic lines of Cd (I)228.80 nm, Pb (I) 405.78 nm and Cr (I) 427.48 nm were selected for further analysis.

### 2.3. Optimizing the Experimental Parameters

To obtain the best performance in the detection process, the agarose-to-solution ratio (M_agarose_/V_solution_, g/mL), delay time and the laser pulse energy were optimized. The agarose-solution ratio determines the strength of the hydrogels and films, which, in turn, affects the signal stability during LIBS detection. Laser-induced plasma can last only a very short time. In the primary stage of plasma, the intensity of the atomic emission spectra and the continuum background spectrum have strong signals under the excitation of the pulsed laser. However, as the delay time increases, they will weaken but at different rates, which leads to a change in the signal-to-background ratio (SBR). Signal intensity can also be affected by laser intensity, which directly influences the ablative quantity of the sample.

#### 2.3.1. Optimizing the Agarose-to-Solution Ratio

The agarose-to-solution ratio plays an important role in the characteristics of agarose hydrogels. In general, the higher the proportion of agarose added to the solution, the greater strength of the agarose hydrogel and, accordingly, the greater the thickness and strength of the agarose films obtained after drying. Hence, we investigated the effects of five agarose-to-solution ratios (M_agarose_/V_solution_ from 0.015 to 0.035) on the signal intensity and signal stability of LIBS detection for the samples. 

**Figure 4:**
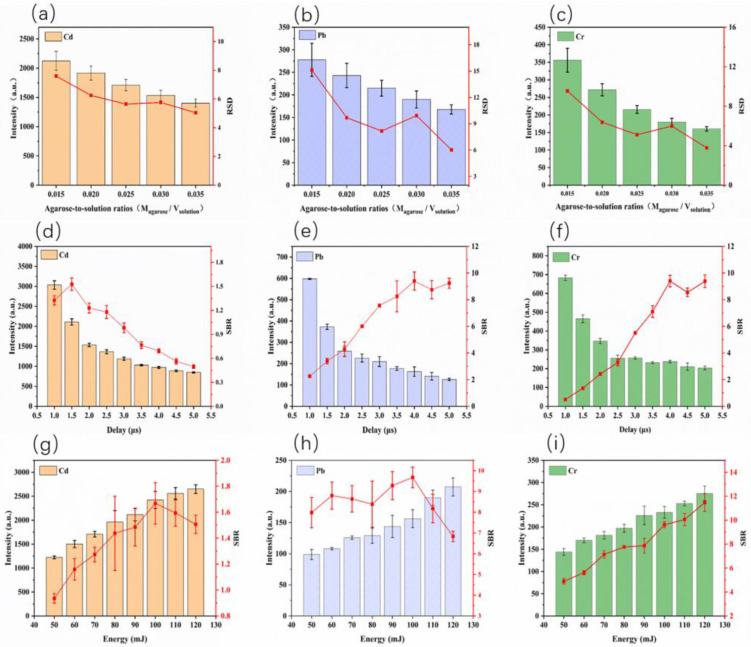
(**a**–**c**) Effects of the agarose-to-solution ratio (M_agarose_/V_solution_) on the spectral intensity and RSD (red line) in the range of 0.015–0.035 (delay time: 2.0 μs and laser pulse energy: 90 mJ). (**d**–**f**) Effects of the delay time on the spectral intensity and SBR (red line) in the range of 1–5 μs (agarose-to-solution ratio M_agarose_/V_solution_: 0.025 and laser pulse energy: 90 mJ). (**g**–**i**) Effects of the laser pulse energy on the spectral intensity and SBR (red line) in the range of 50–120 mJ (agarose-to-solution ratio M_agarose_/V_solution_: 0.025, delay time: 1.5 μs for Cd, and delay time: 4.0 μs for Pb and Cr). The error bars are the standard deviations of the three parallel samples.

We set the delay time to 2.0 μs and the laser pulse energy to 90 mJ. Relative standard deviation (RSD) was used to evaluate the stability. In Figure 4a–c, with the increase of the agarose-to-solution ratio, the emission intensity gradually decreased, likely because the heavy metal ions were diluted as the thickness of the agarose film increased. 

From the general trends, the RSD of the three elemental signals gradually decreased with increasing agarose-to-solution ratio. At low agarose-to-solution ratios, the films were more brittle, and, when subjected to the laser pulse ablation, the ablation points were susceptible to fluctuation. At high agarose-to-solution ratios, due to the formation of thicker films, the strength of the agarose films was enhanced, resulting in an improving coupling stability between the laser pulse and the target elements. To balance the signal intensity and signal stability at the same time, we considered a ratio of 0.025 as the optimized parameter for the further experiments.

#### 2.3.2. Optimizing the Delay Time

When the laser ablates the sample surface, the plasma generated, the gradual cooling, and the kinetic energy of the free electrons decreases after colliding with the ions, and then photons are radiated to form bremsstrahlung radiation. Compound radiation arises when electrons are trapped by ions to form neutral particles and radiating photons. Bremsstrahlung and compound radiation produce continuous spectra and form a continuum background spectrum. 

During the excitation radiation, the electron transition from the high energy level to the low energy level produces a spectrum of specific wavelengths that represent different elements. At the very beginning period of plasma formation, the continuum background spectrum is very strong and dominant. With the plasma cooling, the spectra were dominated by characteristic radiation. 

The delay time is the time between the generation of plasma by laser ablation of the sample and the start of detection of the plasma signal by the detector. The integration time is the detection gate width of ICCD. The sum of the delay time and integration time is approximated as the plasma lifetime. By changing the delay time, a satisfactory SBR can be obtained according to the time evolution law of continuum and characteristic spectra. In this work, the plasma lifetime of the characteristic spectral lines of Cd, Pb and Cr were determined as about 20 μs by pre-experiments. In order to minimize the effects of the continuum background spectrum on the target element peaks, delay time ranging from 1 to 5 μs was investigated, and the gate width changed accompany the delay time. 

We set the agarose-to-solution ratio M_agarose_/V_solution_ to 0.025 and laser pulse energy to 90 mJ. As shown in Figure 4d–f, with the delay time increased, the intensity of Cd, Pb and Cr emissions declined rapidly at the initial period and then leveled off. The SBR of the Cd (I) 228.80 nm spectral line increased until the delay time reached 1.5 μs, and then a gradual reduction was seen. The SBR of the Pb (I) 405.78 nm and Cr (I) 427.48 nm spectral lines continuously increased until the delay time reached 4.0 μs, which meant that, despite the element emission decreasing, the continuum background spectrum intensity decreased more significantly. After comprehensive consideration of the spectra intensity and SBR, the delay times of 1.5 μs for Cd and 4.0 μs for Pb and Cr were selected in the following experiments.

#### 2.3.3. Optimizing the Laser Pulse Energy

Laser pulse energy is an important parameter of LIBS that has a great influence on the ablation of samples and the formation of plasma. Due to the presence of the sample ablation threshold, too low laser pulse energy can lead to instability of the generated plasma. In order to determine the appropriate pulse energy of the laser, an optimizing procedure in the range of 50–120 mJ was performed. We set the agarose-to-solution ratio M_agarose_/V_solution_ to 0.025 and delay time to 1.5 μs for Cd and 4.0 μs for Pb and Cr. 

As clearly shown in Figure 4g–i, the intensity of the three elements gradually increased with the growing laser pulse energy. Owing to the rise of energy, the ablation quantity by single laser pulse also increased. However, when the laser pulse energy increases to a certain level, the intensity growth of the spectral lines slowed down, while the background signal was still enhancing. That is why the SBR of Cd and Pb reached extreme values at 100 mJ and then began to decline. To perform the analysis of the elements simultaneously, 100 mJ was selected as the optimal laser pulse energy.

### 2.4. Quantitative Analysis

**Figure 5:**
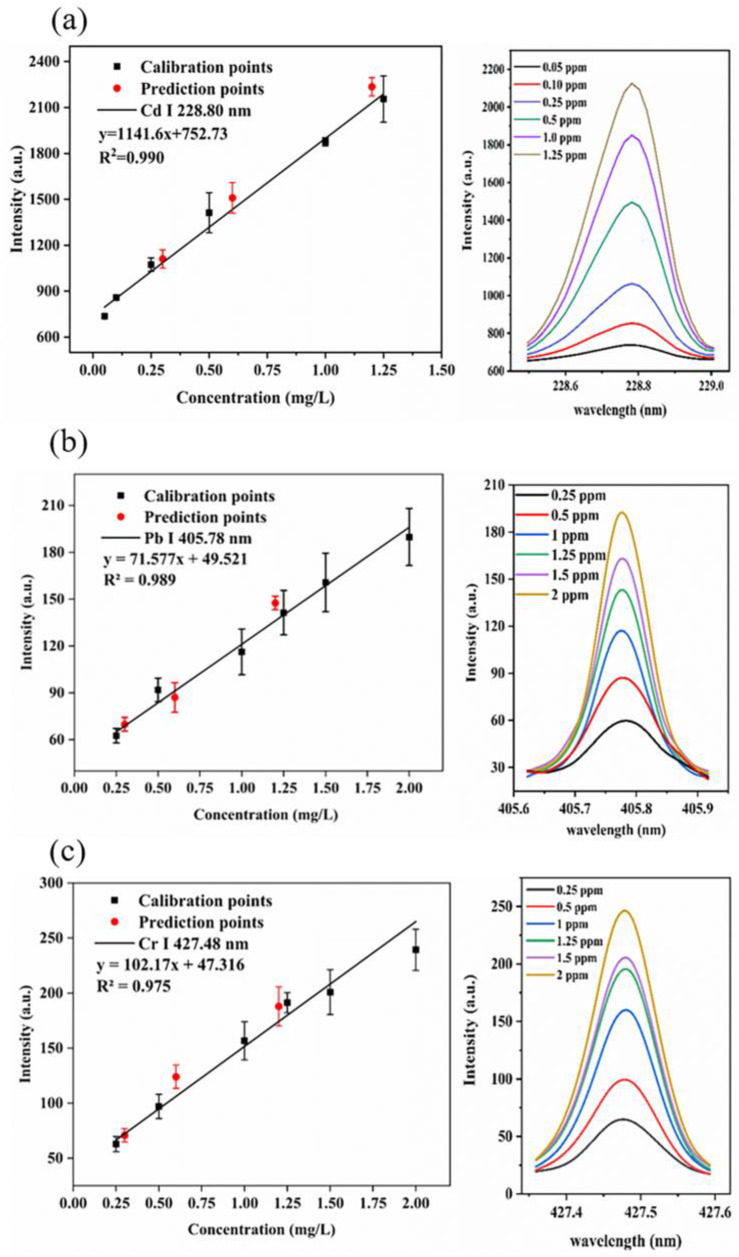
(**a**) Calibration curve of Cd (I) 228.80 nm. (**b**) Calibration curve of Pb (I) 405.78 nm. (**c**) Calibration curve of Cr (I) 427.48 nm. The error bars are the standard deviations of four replications. The right pictures are the spectral intensity of samples with different elemental concentrations in solution.

Calibration curves were established by six standard solutions in the concentration range from 0.05 to 1.25 mg·L^−1^ of Cd and from 0.25 to 2.0 mg·L^−1^ of Pb and Cr under the above-mentioned optimal parameters. Figure 5a–c shows the calibration curves of Cd (I) 228.80 nm, Pb (I) 405.78 nm and Cr (I) 427.48 nm. The error bars at each point reflect the standard deviation of four parallel measurements. Linear regression was used to calculate the regression equations, and the coefficient of determination was used to evaluate the degree of correlation between the emission intensity and the element concentration. The calibration curves of the target elements show a good linear relationship in the fitted concentration range with R^2^ values above 0.97 for all the analytes.

The limits of detection (LOD) for the three elements were calculated according to the equation:

Limit of detection (LOD) = 3σ_B_/s (1)

where σ_B_ represents the standard deviation of background intensities obtained by blank samples, and s represents the slope of the calibration curve. The LODs were 0.011, 0.122 and 0.118 mg·L^−1^ for Cd, Pb and Cr, respectively.

The root-mean-square-error (RMSE) of validation represents the difference between the predicted value and the reference value, expressed as:

(2) $$\mathrm{RMSE}=\sqrt{\frac{\sum_{i=1}^{n}{\left({\hat{y}}_{i}-y_{i}\right)}^{2}}{n}}$$

where $y_{i}$ is the reference concentration of sample i, ${\hat{y}}_{i}$ is the predicted concentration of sample i, and n is the number of samples.

Samples with the heavy metal concentrations of 0.3, 0.6 and 1.2 mg·L^−1^ were prepared to validate the calibration curves. The predicted values of heavy metal concentration were calculated using the calibration curves. The RMSEs of validation were 0.068, 0.107 and 0.112 mg·L^−1^ for Cd, Pb and Cr, respectively.

Various analytical methods have been reported to the literature for the quantification of Cd, Pb and Cr. A comparison of the LODs obtained from the present study with those reported in the literature is presented in Table 1, which can help us to better understand the advantages of the agarose film-based liquid–solid conversion method compared with the general method. After the removal of water, the target elements were pre-concentrated, and the signals were enhanced. The LODs of the new method were lower than most liquid-to-solid methods, such as the hydrogel-based methods, which were apparently affected by the water during the laser pulse ablation. Even when compared with some liquid–solid conversion methods that require expensive materials, such as ion exchange membranes, our method showed competitiveness.

Natural water samples (Qizhen Lake, Zhejiang University, Hangzhou, China) were used to verify the application of the agarose film-based liquid–solid conversion method. Before detection, the lake water samples were filtered through a 0.22 μm membrane to remove insoluble impurities. The lake water was spiked by heavy metal solutions to meet the needs of analysis. The samples were analyzed in triplicate under the optimal experimental conditions.

The recovery value can be mathematically calculated according to the equation:

(3) $$R=\frac{{\bar{x}}^{\prime }-\bar{x}}{x_{spike\;}}\times 100$$

where ${\bar{x}}^{\prime }$ represents the mean value of the concentration found in the spiked sample, $\bar{x}$ represents the mean value of the concentration found in the unspoked sample, and $x_{spike\;}$ represents the added concentration.

Table 2 shows the analytical results of heavy metals in real water samples. The mean recovery values of the spiked samples were in the range of 91.2–107.9%. Generally speaking, the results of the LIBS analysis were satisfactory.

Overall, agarose film-based liquid–solid conversion LIBS is significantly faster in heavy-metal detection in water than chemical methods, such as AAS and ICP-MS. In addition, the proposed method is more environmentally friendly as no chemical contamination (e.g., nitric acid) is introduced during the process with the treatment. For the LIBS method in this study, the drying time can be further shortened, and the detection efficiency can be improved in the future by optimizing the drying parameters, such as the temperature and air speed, or by using advanced drying devices.

## 3. Materials and Methods

### 3.1. Heavy Metal Solution Preparation

Stock solutions (1000 mg L^−1^) of Cd, Pb and Cr were prepared by dissolving known amounts of Cd(NO_3_)_2_·4H_2_O, Pb(NO_3_)_2_ and Cr(NO_3_)_3_·9H_2_O in ultrapure water (18.25 MΩ cm^−1^) and stored in the dark. All the reagents used were of analytical grade chemicals (Sigma-Aldrich, Shanghai, China). Appropriate dilutions were made from the stock solutions to prepare the samples with Cd, Pb and Cr concentrations in the range of 0.05–1.25 mg L^−1^ (Cd), 0.25–2 mg L^−1^ (Pb and Cr) to establish the calibration curves. The agarose powder used was purchased from Aladdin reagent (Shanghai, China), biochemical grade, with the chemical formula C_12_H_18_O_9_ and molecular weight 306.23.

### 3.2. Sample Preparation

**Figure 6:**
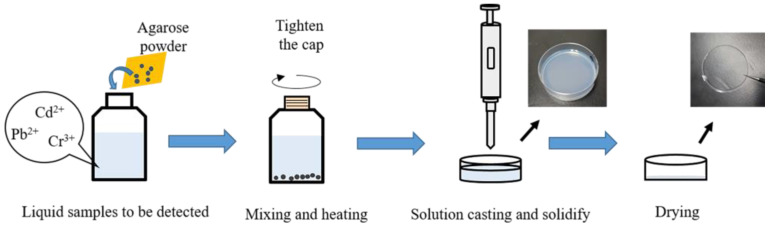
The preparation steps of the agarose hydrogel and agarose film samples.

Using the heavy metal solution samples mentioned above, the preparation steps of the samples are shown in Figure 6.

(1) Weighing 0.125 g of agarose powder with an electronic balance and adding to a screw cap reagent bottle containing 5 mL of standard heavy metal solution.
(2) Screwing the reagent bottle tightly and placing it in the water bath heater for 15 min at 100 °C in order to make sure the agarose is completely dissolved.
(3) Transferring 3.0 g of the solutions into a 3.5 cm diameter petri dish and placing the dishes at room temperature cooling for 5 min to transform the solution into the agarose hydrogel with a smooth surface and homogeneous inside. A portion of the agarose hydrogel samples was kept as a reserve.
(4) Placing the rest agarose hydrogel samples in a ventilated environment until the water completely evaporates.

At this point, the agarose hydrogel and agarose film samples containing heavy metals were obtained. For comparison, blank samples were also prepared under the same operation using ultrapure water instead of a heavy metal solution with an equal mass of agarose.

### 3.3. Experimental Setup

**Figure 7:**
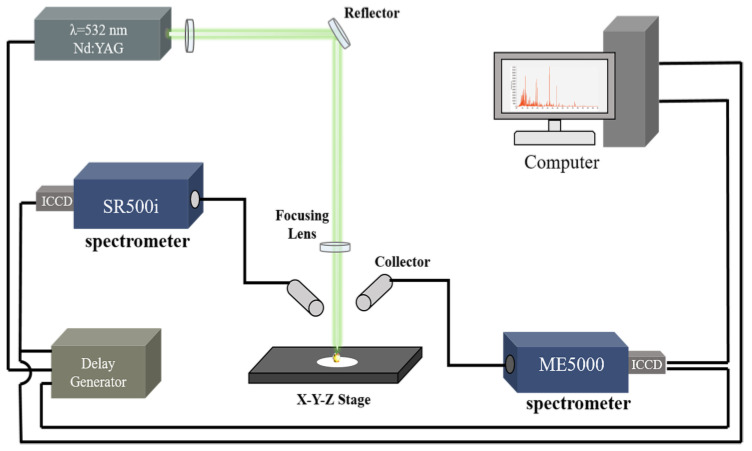
Schematic diagram of the LIBS experimental setup.

The experimental setup of the LIBS system is shown in Figure 7, of which detailed information can be found in article [40]. The excitation source was a Q-switched Nd:YAG nanosecond pulsed laser (Vlite-200, Beamtech Optronics, Beijing, China) operating at 532 nm with a pulse frequency of 1 Hz and a pulsed duration of 8 ns. With the self-designed optical path system, the laser beam passed through a half-wave plate, reflected by a mirror and focused onto the sample 2 mm below the surface by a plano-convex lens (f = 100 mm). 

Under the action of laser ablation, the plasma was excited, and the spectra were collected before the plasma disappeared using the spectrometers, which split the spectrum and obtain high-resolution spectral information. In this study, two spectrometers were used to achieve simultaneous detection of Cd, Pb and Cr. The difference between those two spectrometers is that the spectrometer (ME5000, Andor, Belfast, UK) could split a spectrum with a range from 229 to 878 nm and with a resolution of 0.03 nm for Pb and Cr detection, while the spectrometer (SR-500i-A-R, Andor, Belfast, UK) could split a spectrum in the short wavelength band of 210–231 nm with higher resolution for Cd detection. 

The spectra were converted into electrical signals by an intensified charge-coupled device (ICCD, iStar DH334T-18F-03, Andor, Belfast, UK) camera and recorded in a computer. The digital delay generator (DG645, Stanford Research Systems, -California, USA) enabled realize time series control between the lasers and ICCD detectors. To make the ablation point cover the sample surface and obtain better ablation, an X–Y–Z motorized stage (Zolix, Beijing, China) with movement precision of 0.625 μm was applied to realize the movement of samples, and the spectra were collected at 64 different locations on each sample.

To improve data stability, 64 different positions in a sample were ablated. Each position was ablated once. The spectra were averaged to represent the LIBS spectrum of a sample. The delay time, gate width and pulsed laser energy were the three important parameters for the LIBS system and were optimized.

In this study, a shape measurement laser microscope system (Keyence VK-X3000, Osaka, Japan) was applied to obtain the ablation crater morphology and parameters of samples. SEM used in the experiment was the Phenom ProX system (Phenom ProX, Phenom-World BV, Eindhoven, The Netherlands), which was a platform that integrated scanning electron microscope (SEM) and energy dispersive X-ray spectrometer (EDS) capable of surface microscopy of nano and sub nano-scale samples less than 100 nm in diameter at magnifications up to 100,000 times.

## 4. Conclusions

In this study, we proposed a novel, easy-to-operate and sensitive liquid–solid conversion method using LIBS for detecting heavy metal in water samples. Water samples were transformed into hydrogels to avoid splashes and ripples during the laser ablation process. In order to reduce the effects of moisture on the LIBS determination and enrich the concentration of target elements, agarose hydrogels were dried into agarose films, which resulted in higher signal intensity compared with agarose hydrogel samples and significantly improved the sensitivity of detected elements. The main parameters affecting this method were optimized. 

The calibration curve of the method was built using the optimal experimental parameters. The R^2^ values of the calibration curves were over 0.97, and the LOD values of Cd, Pb and Cr were 0.011, 0.122 and 0.118 mg L^−1^, respectively. The proposed method was successfully validated by standard heavy metal solutions and real water samples. The RMSEs of validation were 0.068 (Cd), 0.107 (Pb) and 0.112 mg·L^−1^ (Cr), and the recovery values were in the range of 91.2–107.9%. Therefore, the presented results demonstrate that agarose film-based liquid–solid conversion is suitable for the detection of Cd, Pb and Cr in water samples. Inexpensive material, simple pretreatment procedures and good LODs make this method promising for heavy metal monitoring in water.

## Figures and Tables

**Table 1:** Comparison of the limit of detection (LOD) of Cd, Pb and Cr using different techniques for water samples.

Spectrum (nm)	Sample Methodology	Matrix Material	LOD (mg L^−1^)	Ref.
Cd (II) 214.44	Ice solidification	Water	1.4	[35]
Cd (II) 214.44	Chelating resin enrichment	Chelating resin	0.036	[25]
Cd (I) 226.50	Biomimatic array droplets	Glass with super hydrophobic surface	0.0135	[26]
Cd (I) 228.80	Agarose film-based liquid–solid conversion	Agarose film	0.011	This work
Pb (I) 405.78	Filter papers absorption	Filter papers	2.7	[36]
Pb (I) 405.78	Ion exchange polymer membranes filtration	Ion exchange polymer membranes	1.1	[37]
Pb (I) 405.78	3D nano-channel porous membrane absorption	3D nano-channel porous membrane	0.081	[38]
Pb (I) 405.78	Agarose film-based liquid–solid conversion	Agarose film	0.122	This work
Cr (I) 425.43	Hydrogel based solidification	Sodium poly acrylate resin	4.44	[23]
Cr (I) 425.43	Ion exchange polymer membranes filtration	Ion exchange polymer membranes	0.46	[39]
Cr (I) 427.48	3D nano-channel porous membrane absorption	3D nano-channel porous membrane	0.11	[38]
Cr (I) 427.48	Agarose film-based liquid–solid conversion	Agarose film	0.118	This work

**Table 2:** Concentrations and recovery values of Cd, Pb and Cr in spiked lake samples.

Element	Spiked (mg·L^−1^)	Found Value (mg·L^−1^)	Recovery (%)
Cd	0	ND ^a^	/
	0.6	0.621 ± 0.082	103.50%
Pb	0	ND ^a^	/
	0.6	0.547 ± 0.078	91.20%
Cr	0	ND ^a^	/
	0.6	0.647 ± 0.027	107.90%

^a^ ND: not detected.

## Data Availability

The data presented in this study are available on request from the corresponding author. The data are not publicly available due to privacy.

## Sample Availability

Samples of the compound are not available from the authors.
